# Study of the Microstructure and Cracking Mechanisms of Hastelloy X Produced by Laser Powder Bed Fusion

**DOI:** 10.3390/ma11010106

**Published:** 2018-01-11

**Authors:** Giulio Marchese, Gloria Basile, Emilio Bassini, Alberta Aversa, Mariangela Lombardi, Daniele Ugues, Paolo Fino, Sara Biamino

**Affiliations:** Department of Applied Science and Technology, Politecnico di Torino, Corso Duca degli Abruzzi 24, 10129 Torino, Italy; gloria.basile@polito.it (G.B.); emilio.bassini@polito.it (E.B.); alberta.aversa@polito.it (A.A.); mariangela.lombardi@polito.it (M.L.); daniele.ugues@polito.it (D.U.); paolo.fino@polito.it (P.F.); sara.biamino@polito.it (S.B.)

**Keywords:** Ni-based superalloys, Hastelloy X alloy, laser powder bed fusion, electron microscopy, microstructure, XRD analysis, carbides

## Abstract

Hastelloy X (HX) is a Ni-based superalloy which suffers from high crack susceptibility during the laser powder bed fusion (LPBF) process. In this work, the microstructure of as-built HX samples was rigorously investigated to understand the main mechanisms leading to crack formation. The microstructural features of as-built HX samples consisted of very fine dendrite architectures with dimensions typically less than 1 µm, coupled with the formation of sub-micrometric carbides, the largest ones were mainly distributed along the interdendritic regions and grain boundaries. From the microstructural analyses, it appeared that the formation of intergranular carbides provided weaker zones, which combined with high thermal residual stresses resulted in hot cracks formation along the grain boundaries. The carbides were extracted from the austenitic matrix and characterized by combining different techniques, showing the formation of various types of Mo-rich carbides, classified as M_6_C, M_12_C and M_n_C_m_ type. The first two types of carbides are typically found in HX alloy, whereas the last one is a metastable carbide probably generated by the very high cooling rates of the process.

## 1. Introduction

Laser powder bed fusion (LPBF) has the potential to build near net shape components with complex geometries starting from consecutive layers of loose powders, which are directly consolidated by laser beam irradiation, thus adding material layer by layer [1,2,3].

This technology is a type of additive manufacturing process that allows the fabrication of a wide range of metal alloys, reaching high densification levels, such as titanium alloys [3,4], aluminium alloys [5,6,7], stainless steel [8], and different kinds of composites [9] as well as superalloys [10,11,12,13].

In particular, for fabricating materials with complex shapes characterized by high temperature strength such as Ni-based superalloys the LPBF can drastically reduce production costs, taking the place of traditional processes, such as casting and forging [12,14].

In fact, after an adequate process parameters optimization, LPBF enables the production of different Ni-based superalloys with relative density close to 100%, among which the most investigated ones are Inconel 718 and Inconel 625 alloys [10,11,12,13,15]. 

Notwithstanding this, for Ni-based superalloys, characterized by high crack susceptibility, the occurrence of cracks is difficult to avoid, which reduces the densification level, even when a process parameter optimization is applied [15,16].

One of these Ni-based superalloys is represented by Hastelloy X (hereafter abbreviated as HX), which is a solid solution hardening alloy, combining high-temperature strength and exceptional oxidation resistance. All these properties make it an excellent material to build parts of rocket engines, gas turbine engines for combustion zone components such as transition ducts, combustor cans as well as chemical process industry [14,17,18,19,20].

In particular, HX components are typically used after a solution annealing at 1175 °C, generating a microstructure with equiaxed grains and primary Mo-rich carbides. Short thermal exposures promote the carbides formation, Mo-rich carbides (M_6_C or M_12_C) and Cr-rich M_23_C_6_ carbides, based on the temperatures and times. On the other hand, detrimental phases such as µ and σ are formed for prolonged thermal exposures [21,22].

The first studies on LPBF HX attempted to determine the specific process parameters in order to eliminate cracks, establishing that it was not possible to obtain crack-free components [17,19]. Afterwards, much research on LPBF HX alloy has been focused on chemical composition modifications in order to reduce cracks, which are typically classified as hot tearing or hot cracking, occurring during the last period of solidification [14,18,23,24].

Harrison et al. [14] suggested that cracks occur when thermal residual stresses overcome the ultimate tensile strength (UTS) of the alloys at a precise point and temperature. Therefore, they proposed that it is possible to reduce the number of hot cracks by improving the UTS, which can be obtained by adding more solid solution strengthening elements, such as Cr, Mo, and W. In fact, they pointed out that a modified HX alloy enrichment in solid solution strengthening elements involved less cracks than original HX alloy composition.

Other works of Dacian et al. [23,24] reported that lower concentrations of Si, Mn, and C can result in minor formation of microsegregation along the grain boundaries, consequently generating less cracks. In particular, using a computational thermodynamics approach, in a subsequent work Dacian et al. [18] proposed that the cracking mechanism is mainly influenced by the quantity of Si and C whereas Mn has a negligible effect. It seems, in fact, that during solidification Si and C have a remarkable impact on the hot cracking sensibility, increasing the concentration of the eutectic phase and decreasing the solidification range. They underlined that cracks occur due to the formation of M_6_C carbides promoted by C and Si, as well as σ phase along the grain boundaries coupled with high thermal residual stresses. Similarly, for another kind of Hastelloy alloy, it was reported that Si increases the amount of M_6_C carbides along the grain boundaries, providing a broad supply of crack initiation [25].

Based on these considerations, in this work an in-depth microstructural analysis of as-built HX of a non-modified chemical composition was performed, revealing the presence of carbides scattered throughout the materials, also along the cracks thus providing distinct evidence of the role of these carbides in cracking mechanisms. Finally, regarding the very fine size of these carbides, we succeeded in extracting them from the austenitic matrix of the samples, in order to study their nature by combining different analyses.

## 2. Materials and Methods 

### 2.1. Powder Characterization

The chemical composition of the powder feedstock and processed LPBF solid samples were assessed using an inductively coupled plasma-optical emission spectroscopy (ICP-OES) analysis, except for C and O_2_ evaluated by means of an infrared absorption (IRA) and an inert gas fusion (IGF) analysis, respectively. The determined chemical compositions are listed in Table 1 and present values within the allowable tolerances reported in the ASTM B435-06. Additionally, the very low concentration of O_2_ within the powder and solid HX samples indicates that no contamination occurred during the LPBF process.

The gas atomized HX powders were characterized using a field emission scanning electron microscope (FE-SEM, Zeiss Merlin, Oberkochen, Germany) and scanning electron microscope (SEM, Phenom XL, Phenom-World BV, Eindhoven, The Netherlands), in order to observe their morphology. Moreover, the powder was mounted, ground and polished down to 0.05 µm using Al_2_O_3_ suspension, in order to determine the residual porosity within the particles by means of an optical microscope (OM, Leica DMI 5000 M, Wetzlar, Germany). The average value and standard deviation of the residual porosity were determined using 200 particles at a magnification of 500×. Laser granulometry (Fritsch model Analysette 22 Compact, Fritsch, Idar-Oberstein, Germany) was employed to determine the cumulative particle size distribution of the powder. Finally, the apparent density and flow rate were assessed following the ASTM B212-13 and ASTM B213-13 standards, respectively.

### 2.2. LPBF Hastelloy X Specimens: Production and Geometrical Features

The HX specimens were fabricated from gas atomized HX powders supplied by LPW Technology (LPW Technology Ltd., Runcorn, UK) using an EOSINT M270 Dual Mode version (EOS GmbH, Munich, Germany) laser powder bed fusion machine, equipped with a Ytterbium 200 W fiber laser. Note that the process parameters are omitted to protect the proprietary information.

**Figure 1:**
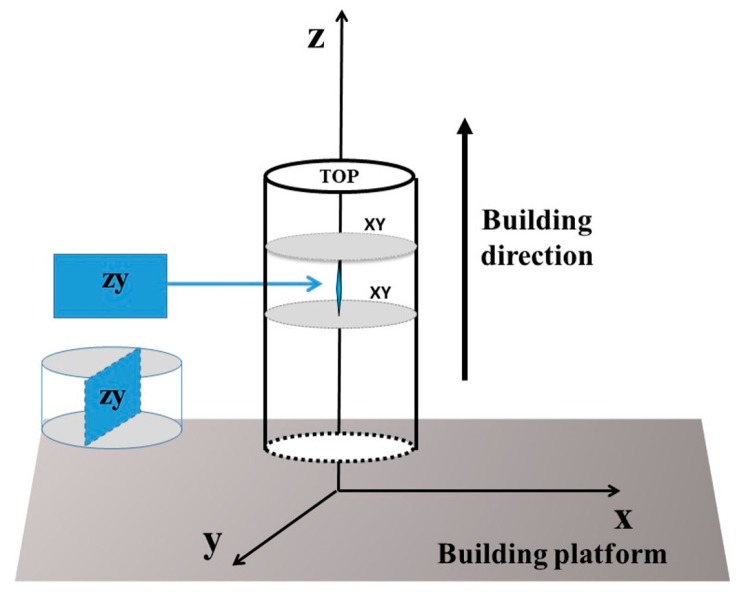
Schematic representation of the as-built HX cylinders built along the building direction; the x-y plane is perpendicular to the building direction, while the z-y plane is parallel to the building direction.

Cylindrical samples with a length of 77 mm and a diameter of 14 mm were built along the building direction (*z*-axis). The as-built HX specimens were cut into smaller samples with a length of 5 mm, and then the microstructure was investigated along the x-y plane, which is perpendicular to the building direction, and along the z-y plane, which is parallel to the building direction, as schematized in Figure 1.

### 2.3. Microstructural Investigation

The as-built HX samples were ground using SiC grinding paper (1200 and 2400 grit sizes), and polished up to 1 µm using diamond suspension. The polished samples were analyzed by taking 20 OM images at 100×, in order to determine the residual porosity and cracking density by post-process analysis with Image J. All the OM images allowed a total surface coverage of 13.2 mm^2^ for each HX state. For the measurement of the cracking density, the feret length was considered and the values were reported in the unit “mm of microcracks per mm^2^ of OM image”, as stated in other studies on Ni-based superalloys with cracks [15,16].

The HX polished samples were subsequently etched using Kalling’s No.2 reagent (5 g CuCl_2_ in 100 mL HCl and 100 mL CH_3_CH_2_OH) in order to reveal microstructural features such as grains, melt pools and carbides. For this purpose, the etched HX samples were observed by means of OM, as well as FESEM and SEM both equipped with an Energy Dispersive X-ray Spectroscopy (EDS) detector. 

For the as-built HX samples, an adequate amount of carbides were anodically extracted at 2 V using a solution of 25% HCl–75% CH_3_OH at room temperature, following a similar procedure described in literature [25]. The apparatus consisted of a beaker to contain the solution, a dc power supply to provide 2 V, a stainless-steel cathode and the HX samples as an anode. In this way, the matrix of the HX sample tends to dissolve within the solution, making possible to collect the carbides.

Afterwards, the extracted carbides were investigated through FESEM + EDS, SEM + EDS and X-ray diffraction (XRD) analyses, in order to identify the types of formed carbides. XRD analysis was carried out by using an X-Pert Philips diffractometer (PANalytical, Almelo, The Netherland) by CuKα radiation in a Bragg Brentano configuration working at 40 kV and 40 mA with a step size of 0.013° and a counting at each step for 35 s, recording the XRD data from 30° to 65°.

## 3. Results and Discussion

### 3.1. Powder Characterization

**Figure 2:**
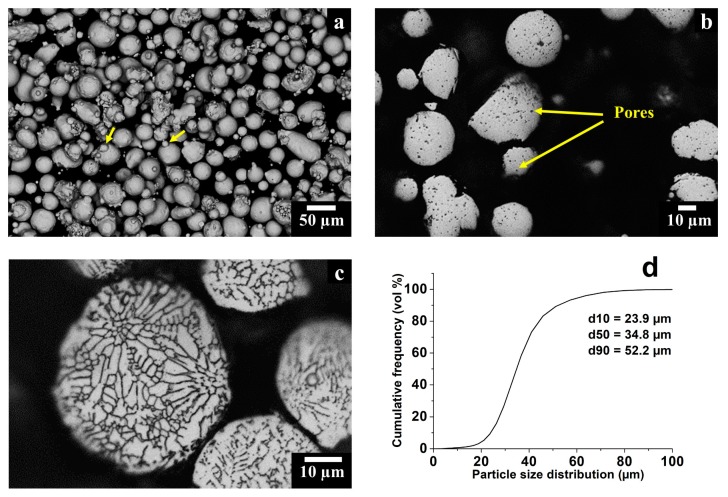
(**a**) SEM image in backscattered electron (BSE) mode of gas atomized HX powder showing spherical and some irregular particles together with some satellites indicated by yellow arrows; (**b**) OM image of HX particles cross section showing fine spherical pores; (**c**) OM image of HX particles cross section exhibiting fine dendritic structures; (**d**) Particle size distribution HX powder.

The starting gas atomized HX particles were generally spherical, exhibiting some irregular and satellites particles. The latter ones are formed during the atomization process due to particle collisions, indicated by yellow arrows in Figure 2a. The mounted and polished particle cross section, in Figure 2b, allowed the observation of near-perfect spherical pores, of which the largest ones had a diameter of around 5 µm, evaluating a residual porosity of 1.50 ± 0.30%. The formation of these spherical pores may stem from the entrapped gas during the atomization process [26]. Also, the mounted and polished particles were etched with Kalling’s No. 2 reagent revealing very fine dendritic structures derived from the gas atomization process, as displayed in Figure 2c. The particle size distribution had a d10 of 23.9 µm, a d50 of 34.8 µm and a d90 of 52.2 µm, as illustrated by the cumulative frequency of particle size distribution in Figure 2d. Finally, the average apparent density for the LPBF powder was 3.98 ± 0.02 g/cm^3^, whereas the flow rate determined by Hall flowmeter was 13.6 ± 0.4 s/50 g.

### 3.2. Cracking Density and Residual Porosity Investigation

**Figure 3:**
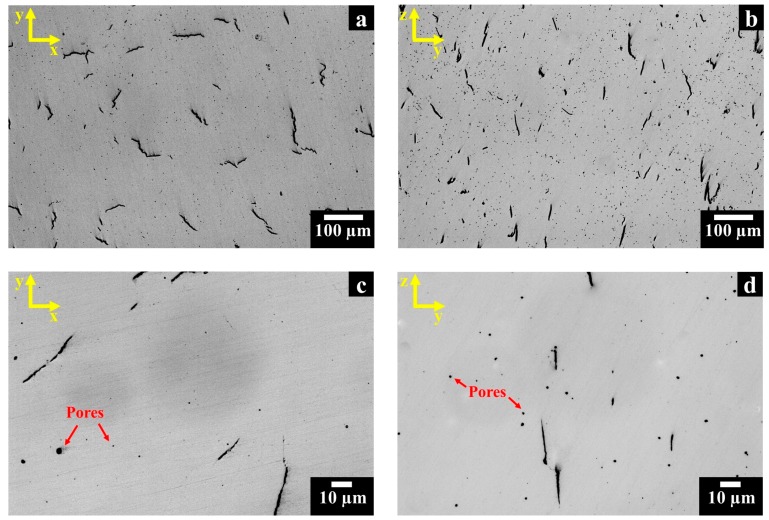
OM images of polished as-built HX samples showing: (**a**,**b**) cracks and pores along the x-y and z-y planes, respectively; (**c**,**d**) higher magnification exhibiting the near-perfect spherical shape of the micrometric pores along the x-y and z-y planes, respectively.

The OM images of polished as-built HX samples along the x-y and z-y planes are shown in Figure 3. The as-built HX samples revealed several cracks with a length up to 100–150 µm, randomly distributed in the x-y plane (Figure 3a), while they were mainly located along the building direction in the z-y plane (Figure 3b).

As seen at higher magnification, both along the x-y plane (Figure 3c) and the z-y plane (Figure 3d), the microstructure exhibited spherical pores with diameters from sub-micrometric size to approximately 5 µm. The size and shape of the pores suggest that they may arise from the entrapped gas within the starting powder, observed in Figure 2b [2,27].

Table 2 summarizes the average value and standard deviation of residual porosity and cracking density of as-built HX samples along the x-y and z-y planes. The calculated cracking density and residual porosity values are in line with the cracking density reported in other studies on LPBF HX alloys [14,17].

### 3.3. Microstructural Investigation of As-Built Hastelloy X Samples

**Figure 4:**
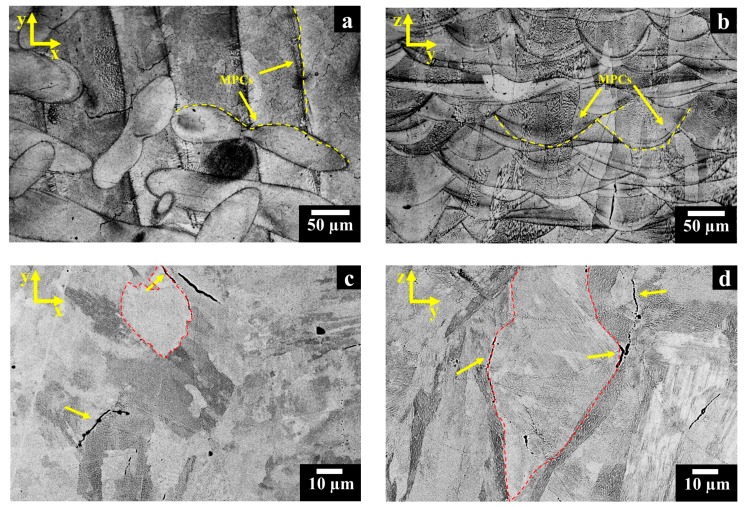
(**a**,**b**) OM images of as-built Hastelloy X (HX) sample showing melt pool contours (MPCs) along the x-y and z-y planes; (**c**,**d**) SEM images in BSE mode of as-built HX sample with equiaxed (x-y plane) and columnar (z-y plane) grains marked by red dash lines, exhibiting cracks crossing the grain boundaries, as indicated by yellow arrows.

The microstructures of as-built HX samples revealed traces of melt pool contours (MPCs) with different shapes along the x-y and z-y planes, generated by the laser beam during the LPBF process, as highlighted by the yellow dash lines in Figure 4a,b, respectively. In the current work, the melt pools are not aligned according to the EOS laser scanning strategy, in which the laser beam is rotated by 67° before melting the subsequent layer of loose powder [10,28].

The SEM images display randomly equiaxed grains along the x-y plane (Figure 4c) as well as columnar grains along the z-y plane (Figure 4d), and grain boundaries are highlighted by red dash lines. The formation of equiaxed and columnar grains along different planes have been widely reported in several works on LPBF Ni-based superalloys, indicating the columnar grains derived from the heat flux dissipation from the top of the material to the building platform [28,29,30]. As can be seen in the SEM micrographs, the cracks mostly lay along the equiaxed (x-y plane) and columnar (z-y plane) grains, as indicated by the yellow arrows in Figure 4c,d, respectively.

**Figure 5:**
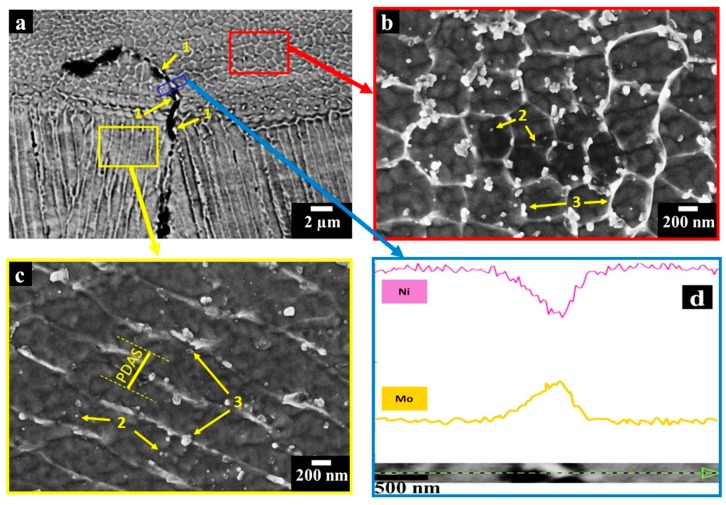
SEM images of HX samples showing (**a**) very fine cellular and columnar dendritic structures as well as an intergranular crack and bright phases indicated by arrows 1, taken in BSE mode; (**b**,**c**) cellular and columnar dendrites with sub-micrometric primary dendrite arm spacing (PDAS) exhibiting very fine Mo-rich carbides within dendritic core indicated by arrows 2 together with globular and elongated carbides pointed out by arrows 3 within interdendritic areas, taken in secondary electron (SE) mode; (**d**) EDS line scan of the bright phases along the cracks showing that the precipitates were enriched in Mo and depleted of Ni.

At higher magnifications (Figure 5a), it is possible to note that the melt pools are made up of extremely fine dendritic architectures with cellular and columnar shapes, as well as the presence of cracks covered by bright phases (indicated by arrows 1). 

The cellular dendrites (Figure 5b) are believed to be altered primary dendrites caused by the inherently high heating and cooling rates of the process, whereas the columnar dendrites (Figure 5c) are clearly primary dendrites [31]. 

The cooling rates (ε) were estimated using the determined primary dendrite arm spacing (PDAS) by FESEM micrographs, as shown in Figure 5c, using Equation (1) [32]:

(1) $$\mathrm{PDAS}=a\;\varepsilon^{-b}$$

where *a* and *b* are material constants, in particular for Ni-based superalloys, *a* ≈ 50 μm (K/s), while *b* = 1/3 [32]. The image analyses assessed PDAS of 0.65 ± 0.25 µm, thus resulting in an average cooling rate of around 4.5 × 10^5^ K/s, revealing good agreement with other studies on Ni-based superalloys produced by the LPBF process [14,32,33]. The cellular and columnar dendrite cores denoted nanometric precipitates with a size typically less than 100 nm, as indicated by arrows 2. In addition, globular and elongated bright phases around 100–500 nm were predominantly observed along the interdendritic regions, pointed out by arrows 3. 

EDS line scans performed on the largest bright phases along the cracks revealed an enrichment in Mo correlated with a depletion of Ni (Figure 5d), indicating the formation of intergranular Mo-rich carbides. The presence of carbides along the intergranular cracks provides evidence that the cracks are triggered by the carbides coupled with the high thermal residual stresses induced by the process. Due to the high residual stresses, also the pores may act as a source for cracks formation. However, the carbides decorate the cracks, thus indicating their main role in the cracking mechanisms.

**Figure 6:**
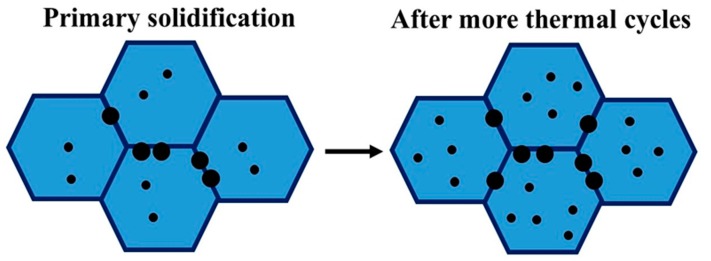
Schematic diagram of cellular dendritic structures of as-built HX samples. The black circle represents the Mo-rich carbides whereas the blue and dark blue areas represent the dendritic and interdendritic regions, respectively.

From the microstructural observations, it is possible to suppose that during solidification the elements with high susceptibility to segregation (i.e., Mo, W, C, Si) are rejected from dendrite to interdendritic regions, as well as along the grain boundaries. Furthermore, the material is subjected to more thermal cycles consisting of several remelting as well as continuous heating and cooling rates, where each cycle may induce the formation of new carbides. This hypothesis on the carbide formation during the primary solidification and then triggered by subsequent thermal cycles in the course of the LPBF process is shown schematically in Figure 6, where the black circles represent the Mo-rich carbides, and the blue and dark blue areas represent the dendritic and interdendritic regions, respectively.

**Figure 7:**
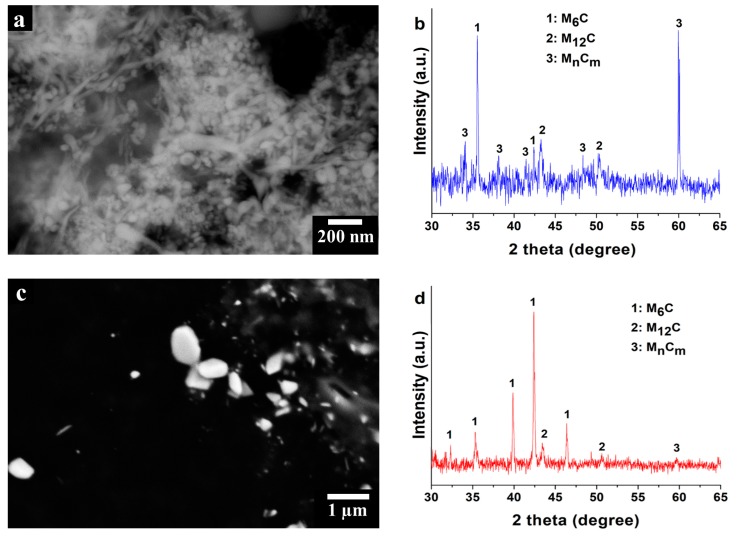
(**a**) SEM image in BSE mode of extracted Mo-rich carbides of as-built HX samples; (**b**) XRD data of extracted carbides from as-built HX samples; (**c**) SEM image in BSE mode of extracted Mo-rich carbides of heat-treated HX samples; (**d**) XRD data of extracted carbides from heat-treated HX samples.

In order to reach a better understanding of the nature of these carbides, they were anodically extracted from the austenitic face-centered cubic (γ-fcc) matrix of HX alloys. The morphology of the extracted carbides (Figure 7a) and the EDS analyses (Table 3) revealed a remarkable enrichment in Mo, W and Si, compared to the γ-fcc matrix, confirming the formation of Mo-rich carbides.

The Mo-rich carbides present in HX alloy are commonly characterized by an fcc structure, so it is relatively easy to distinguish the type of Mo-rich carbides presented within the as-built samples by XRD analysis [21,22,34,35]. Figure 7b shows the XRD data of the extracted Mo-rich carbides revealing several peaks which can be associated with Mo-rich M_6_C, Mo-rich M_12_C (also defined as M_6_C’ [34]) and Mo-rich M_n_C_m_ carbides (“n”, “m” not defined), with lattice parameters (a_o_) of 11.05 ± 0.02 Å, 10.87 ± 0.01 Å and 10.58 ± 0.07 Å. In particular, the intensity of the peaks indicates a major fraction of Mo-rich M_6_C and Mo-rich M_n_C_m_ carbides compared with Mo-rich M_12_C carbides. The formation of Mo-rich M_6_C carbides occurs during the last stages of solidification [35], while both Mo-rich M_6_C and Mo-rich M_12_C are typically found in heat-treated HX alloys [21,22,34].

It is therefore possible to suppose that the very high cooling rates of the LPBF process provoke the formation of metastable Mo-rich M_n_C_m_ carbides, as already proposed by Divya et al. [36], investigating LPBF CM247LC by TEM characterizations. 

These Mo-rich M_n_C_m_ carbides could transform into stable Mo-rich M_6_C or M_12_C carbides during a heat treatment involving atomic diffusion. To further prove the nature of the detected carbides, a heat treatment at 1066 °C for 1 h followed by water quenching was performed. More precisely, the selected heat treatment promotes the formation of Mo-rich M_6_C carbides, based on the temperature-time-transformation (TTT) diagram of HX alloy [22]. The heat treatment was successively followed by water quenching to avoid the generation of new carbides during cooling. 

Morphological (Figure 7c) and EDS analyses (Table 3) of the extracted carbides revealed the presence of Mo-rich carbides, characterized by larger dimensions compared with the as-built extracted carbides.

The XRD data of the extracted carbides from the heat-treated HX samples are shown in Figure 7d. For the heat-treated HX samples, the intensity of the peaks increases for the Mo-rich M_6_C carbides and slightly decreases for the Mo-rich M_12_C carbides compared with those of the as-built HX samples. This implies that the thermal exposure promotes an increment of the quantity of the Mo-rich M_6_C carbides, together with the Mo-rich M_12_C carbides decrement. Furthermore, the heat-treated HX samples show only one small peak of the M_n_C_m_ carbides, revealing a significant decrease of their fraction compared with the as-built state.

These results further support the hypothesis that the inherently high cooling rates of the LPBF process can result in the formation of metastable fcc M_n_C_m_ carbides, which tend to transform into Mo-rich M_6_C carbides only after the application of heat treatments involving atomic diffusion, in order to reach a stable state. 

## 4. Conclusions

The fabrication of LPBF HX components with a complex shape can reduce production costs with respect to traditional processes, even if cracking generally occurs during the LPBF process due to the building parameters or the chemical composition of the powders. 

In this work, an in-depth microstructural characterization of as-built HX samples with standard chemical composition was performed, in order to describe the microstructure as well as offer an understanding of the key elements of the cracking phenomena. 

It was found that the microstructure is composed of columnar grains along the building direction (z-y plane) and equiaxed grains perpendicular to the building direction (x-y plane). The very high heating and cooling rates of the process resulted in fine cellular and columnar dendrite architectures, mainly with dimensions less than 1 µm, as well as the formation of sub-micrometric fcc Mo-rich M_n_C_m_, Mo-rich M_12_C and M_6_C carbides. These carbides are chiefly larger along the grain boundaries and interdendritic areas, reaching sizes up to around 500 nm, compared with the carbides within dendritic cores, with sizes less than 100 nm. These Mo-rich carbides were also detected along the cracks, lending support to their role in the cracking mechanisms. It is therefore possible to assume that a significant number of cracks was generated by the combination of intergranular carbides and high thermal residual stresses of the LPBF process. 

Understanding the cracking mechanism within HX alloys with standard chemical composition provides compelling evidence that chemical composition modification is one of the main approaches to generating crack-free HX components by LPBF.

## Figures and Tables

**Table 1:** Chemical composition of the main elements obtained by inductively coupled plasma-optical emission spectroscopy (ICP-OES), infrared absorption (IRA) and inert gas fusion (IGF) analyses on powder and solid (post-processed) HX samples.

Element (wt %)	Ni	Cr	Fe	Mo	Co	Si	W	C	O_2_
Powder HX	Bal	21.70	18.60	9.20	1.82	0.36	0.90	0.056	0.017
Solid HX	Bal	21.40	18.70	9.35	1.85	0.37	0.92	0.051	0.008

**Table 2:** Residual porosity and cracking density of as-built HX samples along different orientations.

Orientation		As-Built HX
x-y plane	Residual porosity (%)	0.25 ± 0.06
z-y plane	Residual porosity (%)	0.31 ± 0.05
x-y plane	Cracking density (mm/mm^2^)	2.60 ± 0.60
z-y plane	Cracking density (mm/mm^2^)	3.32 ± 0.58

**Table 3:** EDS results in wt % of γ-fcc matrix and extracted Mo-rich carbides of as-built and heat-treated HX samples.

**As-Built HX State**							
**Phase**	**Ni**	**Cr**	**Fe**	**Mo**	**Co**	**W**	**Si**
**γ-fcc matrix**	46.6 ± 0.5	21.6 ± 0.3	17.7 ± 0.3	10.1 ± 0.4	1.9 ± 0.2	1.1 ± 0.3	1.0 ± 0.2
**Mo-rich carbides**	16.8 ± 0.6	22.6 ± 0.5	7.8 ± 0.4	37.4 ± 0.7	0.6 ± 0.3	9.3 ± 0.6	5.5 ± 0.3
**Heat-Treated HX State**							
**Phase**	**Ni**	**Cr**	**Fe**	**Mo**	**Co**	**W**	**Si**
**γ-fcc matrix**	46.9 ± 0.4	21.4 ± 0.3	17.9 ± 0.3	10.1 ± 0.3	1.8 ± 0.3	1.0 ± 0.3	0.9 ± 0.1
**Mo-rich carbides**	11.0 ± 0.5	10.5 ± 0.4	4.4 ± 0.4	56.9 ± 0.6	0.6 ± 0.4	13.3 ± 0.5	3.3 ± 0.3
